# Potential Carcinogens in Makeup Cosmetics

**DOI:** 10.3390/ijerph20064780

**Published:** 2023-03-08

**Authors:** Radosław Balwierz, Paweł Biernat, Agata Jasińska-Balwierz, Dawid Siodłak, Anna Kusakiewicz-Dawid, Anna Kurek-Górecka, Paweł Olczyk, Wioletta Ochędzan-Siodłak

**Affiliations:** 1 Faculty of Chemistry, University of Opole, 45-052 Opole, Poland; 2 Department of Drug Forms Technology, Faculty of Pharmacy, Wroclaw Medical University, 50-556 Wroclaw, Poland; 3 Department of Pharmacology, Academy of Silesia, 40-555 Katowice, Poland; 4 Department of Community Pharmacy, Faculty of Pharmaceutical Sciences in Sosnowiec, Medical University of Silesia in Katowice, Kasztanowa 3, 41-200 Sosnowiec, Poland

**Keywords:** makeup, carcinogens, cancer, ethanolamines, heavy metals

## Abstract

Facial makeup cosmetics are commonly used products that are applied to the skin, and their ingredients come into contact with it for many years. Consequently, they should only contain substances that are considered safe or used within an allowable range of established concentrations. According to current European laws, all cosmetics approved for use should be entirely safe for their users, and the responsibility for this lies with manufacturers, distributors, and importers. However, the use of cosmetics can be associated with undesirable effects due to the presence of certain chemical substances. An analysis of 50 random facial makeup cosmetics commercially available on the European Union market and manufactured in six European countries was carried out, concerning the presence of substances with potential carcinogenic properties, as described in recent years in the literature. Nine types of facial makeup cosmetics were selected, and their compositions, as declared on the labels, were analyzed. The carcinogens were identified with information present in the European CosIng database and according to the Insecticide Resistance Action Committee’s (IRAC) classification. As a result, the following potential carcinogens were identified: parabens (methylparaben, propylparaben, butylparaben, and ethylparaben), ethoxylated compounds (laureth-4, lautreth-7, or ethylene glycol polymers known as PEG), formaldehyde donors (imidazolidinyl urea, quaternium 15, and DMDM hydantoin), and ethanolamine and their derivatives (triethanolamine and diazolidinyl urea), as well as carbon and silica. In conclusion, all of the analyzed face makeup cosmetics contain potential carcinogenic substances. The literature review confirmed the suppositions regarding the potential carcinogenic effects of selected cosmetic ingredients. Therefore, it seems necessary to carry out studies on the long-term exposure of compounds present in cosmetics and perhaps introduce stricter standards and laws regulating the potential presence of carcinogens and their activity in cosmetics.

## 1. Introduction

The skin, as the human body’s largest organ, forms a protective barrier, protecting the body from the effects of microorganisms and physical and chemical agents [1]. At the same time, it has the ability to allow substances to be absorbed into the body that can affect physiological processes and cause adverse effects, including toxic effects [2,3]. Lipophilic molecules without electric charge and with a molecular weight below 500 Da can passively penetrate through the skin, and some factors, such as temperature or skin occlusion, can increase and facilitate the penetration of substances used in cosmetics [4]. Therefore, taking into account the exposure of the skin to makeup cosmetics for many hours, there is a risk of transdermal absorption of some substances, as well as their accumulation in the body. The predisposing factors for cancer, one of the leading causes of death in developed countries, are considered to be the most dangerous [5].

Due to the sharp increase in the incidence and mortality of cancer, knowledge of carcinogens is currently the subject of many studies and papers. According to data from the World Health Organization (WHO), about 30% of cancer diagnoses could be prevented with proper prevention, which includes limiting exposure to potentially oncogenic agents. The data suggest that countries with a high level of development have 2–3 times higher cancer incidence than those that are less developed [6]. The most common cancers are breast (2.26 million cases), lung (2.21 million cases), colon and rectum (1.93 million cases), prostate (1.41 million cases), and skin cancers (with 1.20 million cases) [7].

Facial makeup cosmetics are commonly used cosmetic products that are often applied to the skin, meaning that their ingredients often come in contact with it for many years. Given this fact, they should only contain substances considered safe and in concentrations that comply with current standards. The list of substances not recommended for cosmetic use is constantly being updated and expanded based on new scientific reports. Despite extensive advances in the field of toxicology, the presence of compounds with potentially harmful effects in commercially available products cannot be fully excluded [8]. Ingredients with potential carcinogenic properties should be considered particularly dangerous [5,9,10].

### 1.1. Basic Types of Makeup

Makeup application begins with selecting the appropriate products, such as makeup base preparations, foundations, concealers, setting powders, eye and brow shadows, blushes, highlighters, bronzers, cosmetic pencils, lipsticks, glosses, eyeliners, and mascaras [11,12].

A makeup base (primer) is a product applied to the skin before applying color makeup. The primary task of the base is to even out the surface of the skin and allow the makeup to last longer. The product can also be applied to the eye area and lips, i.e., in the form of an eyeshadow base or lipstick base, respectively [11,12].

A primer is applied directly over a skincare cream or makeup base, while a concealer is applied over the foundation. A special type of foundation called a coloring cream has the properties of a skincare cream and a foundation. In addition to pigments that even out skin tone, primers contain active ingredients that affect the physiology and function of the skin [11,12].

The powder is used to fix makeup and smooth the texture of the skin. It is applied over a skincare cream or foundation and concealer to eliminate facial shine. It absorbs sweat and sebum secreted by glands on the surface of the skin, as these reduce the durability of products applied to the face. In addition, powder fills in small irregularities of the skin, making it easier to apply subsequent cosmetics. Eyeshadow is a type of cosmetic powder with an increased amount of colored pigments. It is applied directly to the eyelids or over a shadow base to model the shape of the eyes and highlight the color of the iris [11,12].

Blushes, highlighters, and bronzers are products designed to give the right color to the cheeks and optically model the shape of the face. Depending on the cosmetic formula used to make them, they are applied over a foundation or powder [11,12].

Cosmetic eyeliners are applied to the eyelids, eyebrows, and lips, with the place of application depending on the color of the product. It is a tool used to contour, highlight, and visually correct the shape. A crayon applied to the lip area is called a lip liner. The use of a lip liner further prevents lip-coloring products from running off [11,12].

Lipsticks and lip glosses are products applied directly to the lips or base under makeup. They are used to even out the redness of the lips, giving them a particular color, texture, type, and degree of shine. Glosses are a separate category of products applied to the redness of the lips, characterized by a high content of light-scattering pigments. In addition to their beautifying properties, they can also contain substances with care properties [11,12].

Cosmetic mascaras are called emulsions of various densities and are used to darken the skin or its appendages. They can be applied to the eyelids, in which case they are called eyeliners, and also to the eyelashes, called mascaras. Mascaras used to lengthen eyelashes contain cellulose or silk fibers in their composition, which coat the surfaces of the eyelashes and appear to lengthen them [11,12].

### 1.2. Safety of Makeup Products

According to current European laws, all cosmetics approved for use must be completely safe for their users, and the responsibility for this lies with manufacturers, distributors, and importers. However, sometimes the use of cosmetics is associated with undesirable effects resulting from the presence of certain chemical substances. This group of substances includes, but is not limited to phthalates, *p*-phenylenediamine, formaldehyde, dioxane, parabens, triclosan, and numerous metals, including heavy metals [13].

The safety and quality of cosmetic products available in the European Union are regulated by Regulation 1223/2009/EC of the European Parliament and of the Council of the European Union from 30 November 2009. According to the current legal standards, both the finished cosmetic product and the raw materials contained therein are subject to safety assessments. This is confirmed by a report containing, among other things, the quantitative and qualitative composition; physicochemical data; microbiological specifications; information on possible use, including the place, area, time, and frequency of application of the cosmetic product; and the toxicological profile of all cosmetic components. However, it is difficult to determine the overall safety of a cosmetic product due to the lack of clear toxicological testing for some of the ingredients, as well as detailed regulations on the origin of the raw materials and their possible contamination [8].

Regulation No. 1272/2008/EC of the European Parliament and the Council of the European Union, dated 16 December 2008, on the classification, labeling, and packaging of substances and mixtures, defines the concept of agents harmful to human health. In particular, it describes the genotoxic properties of substances that can alter or damage human DNA which are specific indicators of their mutagenic effects. In addition, this regulation also defined the concept of carcinogenicity as the ability of a certain agent to cause cancer or increase the likelihood of its development [14].

The classification that determines a substance’s carcinogenic potential is maintained by the IARC, acting on behalf of the WHO. IARC coordinates scientific cancer research, identifies carcinogens, promotes activities aimed at early detection, and publishes monographs that evaluate the evidence of the carcinogenic effects of specific substances. These substances are assigned to specific groups. The first group classifies compounds as having proven human carcinogenicity, the second group indicates probable or possible carcinogenicity, and the third group consists of agents not classified as carcinogenic [15]. According to Regulation 1223/2009 on cosmetic products, the safety of substances classified as carcinogenic, mutagenic, or toxic for reproduction (CMR) should be assessed by taking into account the exposure from all sources (cosmetics, chemicals, food, and medicinal products) [8]. All chemical substances classified as CMR Category 1A, 1B, and 2, in accordance with the law regulation 1272/2008 [14], are automatically banned from use in cosmetics. Nevertheless, by way of exception, they may be used if recognized by the SCCS (Scientific Committee on Consumer Safety) and considered safe for use in cosmetic products or when the product does not have alternatives. The last update of the list of substances took place on 14 December 2021, when the European Union (EU) published Regulation (EU) 2021/2204 [16], which extended the Category 1B CMR substances list in each of the three CMR categories covered by Entries 28–30 of Annex XVII to the REACH Regulation. The latest change reflects the updated classification of CMR substances under Regulations (EU) 2020/1182 and (EU) 2021/849. In total, it includes 39 new entries under Entries 28–30 of Annex XVII to REACH [17]. The EU guidance for overall exposure assessment has been developed in consultation with the scientific committee on consumer safety (SCCS), the European Chemical Agency (ECHA), the European Medicine Agency (EMA), and the European Food Safety Authority (EFSA) [18].

The above regulation also contains a list of colorants, preservatives, and radioprotective substances permitted for use in cosmetics. Particularly noteworthy, in the context of cosmetic safety, is the list of substances banned from use in cosmetic products, as well as the list of substances that may be included in cosmetic products only if certain restrictions are met, including, for example, the manner or place of application. These lists are updated periodically (the data presented in this publication were updated as of 1 July 2022) based on scientific reports. This, in turn, may result in commonly used substances being classified as banned substances [8,15].

### 1.3. The Classification of Carcinogens

Carcinogens can be divided into two groups: genotoxic and non-genotoxic. The classification is based on their mechanism of action [19]. The first group, including genotoxic carcinogens, is represented by formaldehyde, acetaldehyde, and ethylene oxide [20]. The mechanism of action of genotoxic carcinogens is associated with a direct influence on DNA of the target cells. Most of the chemical carcinogens, directly or after xenobiotic metabolism, are responsible for the induction of DNA damage and act as genotoxic substances [21].

It should be emphasized that genotoxic carcinogens are considered to represent risk factors at all concentrations because even one or a few DNA lesions may cause mutations and significantly increase tumor risk [20].

Among the second group, referred to as non-genotoxic carcinogens, the following can be enumerated: parabens, heavy metals (e.g., arsenic and beryllium), 1,4-dichlorobenzene, 17β-estradiol, and cyclosporine [21]. The mechanism of action of non-genotoxic carcinogens is associated with the induction of inflammation, immunosuppression, creation of reactive oxygen species (ROS), and influence on receptors. Heavy metals are responsible for tissue-specific toxicity and inflammatory responses. Cyclosporine represents typical immunosuppressants, whereas 2,3,7,8-tetrachlorodibenzo-p-dioxin plays the role of receptor mediators.

However, it should be highlighted that clear-cut criteria for this classification have not been established due to insufficiencies in the available information concerning the mechanisms of action of non-genotoxic carcinogens. Therefore, future research should explain the subcellular mechanisms of carcinogenesis. These mechanisms are necessary for the facility of a classification based on risk estimation from exposure to potential carcinogens.

In this article, a detailed review of makeup products available on the European market was carried out about the content of substances with potential carcinogenic properties, as described in recent years. A series of 50 cosmetics were randomly selected, and their International Nomenclature of Cosmetic Ingredients (INCI) compositions were evaluated for the content of potential carcinogens. The legislation suggests that potentially carcinogenic substances may not be present in cosmetics or may be allowed within a specific range of concentrations. However, the current data do not consider long-term exposure, which is possible for makeup cosmetics. This work focuses on the analysis of the literature data on exposure to substances present in European cosmetics and the literature on scientific data premises related to heavy metals.

## 2. Methods

### 2.1. Selection of Cosmetics for the Analysis

The analyzed cosmetics were produced in Poland, Germany, Sweden, the United Kingdom, France, and Italy. The composition of the cosmetics was determined based on the International Nomenclature of Cosmetic Ingredients (INCI). The classification of chemical substances was based on Regulation 1223/2009/EC of the European Parliament and of the Council of the European Union on cosmetic products on November 30, 2009 [8], and based on warnings issued by the International Agency for Research on Cancer (IARC) [11], a catalog of ingredients and their functions contained in the European CosIng database [12]. For composition evaluation, makeup base formulations (4), face foundations (5), concealers (5), cosmetic powders (including highlighters, bronzers, and blushes) (13), highlighter (1) (which was not a cosmetic powder), eyeshadows (5), cosmetic pencils (3), lip-tinting products (5), and cosmetic mascaras (9) were selected. The products were chosen arbitrarily, at random, from among the makeup products available on the websites of two (2) beauty supply stores located in Poland.

### 2.2. The Literature Data Review

The literature review was based on reports available in the PubMed and Scopus databases, using the names of the compounds and linking the queries with keywords such as “toxicity”, “cosmetics”, and “cancer”. For the review, an effort was made to select articles that pertained to skin application or inhalation or respiratory exposures, and the mechanism of the toxic action of the components was proposed. Attempts were made to show the potential risks that can result from years of cosmetic application to the skin. Heavy metals, which may contaminate cosmetic products, were also included in this review, as shown in the scientific data [22,23,24,25].

## 3. Results

At least one of the substances with potential pro-carcinogenic effects discussed in this article was found in the composition of at least one analyzed cosmetic. Several classes of compounds with carcinogenic potential have been distinguished, such as ethanolamines, formaldehyde and its donors, parabens, *tert*-butyl compounds, and ethoxylated compounds. The research results are presented in Table 1. In addition, heavy-metal contamination is described because the data [22,23,24,25] show that contamination of cosmetics with arsenic, cadmium, lead, and mercury is possible.

## 4. Discussion

### 4.1. Potentially Carcinogenic Substances in Makeup Products

Safety assessments of the cosmetics included their ingredients, combinations, and finished products. Multidirectional scientific studies, often using modern technologies, are used to assess the effects of substances on the human body. Despite complex research, the harmfulness, as well as the carcinogenic effects of many substances, is not fully understood, and these substances are classified as potentially carcinogenic [8,26]. The data discussed below is also presented in Table 2.

#### 4.1.1. Ethanolamines and Their Derivatives

Ethanolamines (Table 3) is the common name for chemical compounds that are classified as aminoethanol derivatives according to the International Union of Pure and Applied Chemistry (IUPAC) nomenclature. They are colorless, transparent, viscous, hygroscopic, and water-soluble liquids with an odor similar to ammonia. In cosmetics, triethanolamine (TEA), 2,2′,2′′-nitrilotri(ethan-1-ol) as per IUPAC and diethanolamine (DEA), and 2,2′-iminodiethanol as per IUPAC were formerly widely used [27]. According to the International Nomenclature Cosmetic Ingredient (INCI) of cosmetic ingredients, triethanolamine is designated as TEA, and derivatives of diethanolamine as cocamide (DEA). These substances act as emulsifiers. In addition, TEA regulates the pH of cosmetics [8]. Triethylamine was found in four of the analyzed cosmetics (Table 1—Items 29, 34, 42, and 44).

DEA and its salts are included in the list of prohibited substances for use in cosmetic products (Annex II/411 of Regulation 1223/2009/EC) [8]. According to the substance’s safety data sheet, DEA is labeled as hazardous, harmful, and likely to cause organ damage with prolonged exposure [28]. However, other DEA derivatives are allowed, but in limited concentrations, where the DEA content must not exceed 0.5% by weight. TEA, on the other hand, according to the Material Safety Data Sheet, is not classified as a hazardous substance, and Regulation 1223/2009/EC includes it in the list of “substances which may be included in cosmetic products only subject to certain restrictions” (Annex III/62 of Regulation 1223/2009/EC). This regulates, among other things, the maximum permissible concentration in the finished cosmetic, the value of which must not exceed 2.5% by weight, and the maximum contamination of DEA must not exceed 0.5% by weight. This means that despite the ban on DEA, it can occur as an impurity of other chemicals that are components in cosmetics [8,28].

Laboratory animal studies published by the National Toxicology Program (NTP) showed an increase in the incidence of liver tumors and renal toxicity when DEA was administered to the skin of mice. TEA during dermal exposure in mice also increased the incidence of liver tumors, particularly in females [27]. The carcinogenic effects on the human body are not clear and require further study. However, in vitro studies conducted by Sun et al. in 1996, using human skin taken from a donor, showed that ethanolamines can penetrate the skin [86]. Based on the results of analyses by Knaak and colleagues, a mechanism of toxic action was proposed involving the impairment of the phospholipid metabolism, resulting in disruption of cell membrane function and structure [87]. According to a 2012 Cosmetic Ingredient Review (CIR), ethanolamines and, in particular, DEA inhibit choline uptake by liver cells, leading to impaired methylation, which can lead to changes in gene promoter regions and subsequently changes in gene expression, which can eventually lead to cancerous lesions [29]. The formation of nitrosamines from reactions between ethanolamines and certain preservatives, such as nitrites in the presence of gastric acid, is also noteworthy. The IARC, as well as the European Union, placed nitrosamines in the category of potentially carcinogenic substances [29,88]. The mechanism of action of nitrosamines may be based on the generation of genotoxic reactive oxygen species (ROS) as a result of their metabolic changes. By linking the damaging effects of free radicals to gene expression, the processes nitrosamines can affect have been detailed. These include blocking the cell cycle, the occurrence of oxidative stress, the disruption of nucleotide metabolism, and the disruption of DNA repair mechanisms, which, together, can lead to the initiation of apoptosis. At the same time, these changes, due to their association with genotoxicity, may increase the risk of cancerous lesions [30]. In addition, nitrosamines are capable of alkylating DNA through the addition of an alkyl radical to guanine deoxyribonucleic acid, which stimulates nucleotide transamination [88,89]. Lim et al. showed that cosmetic products containing N-nitrosodiethanolamine (NDELA), N-nitrosodiethylamine (NDEA), TEA, and DEA are not a safety concern. However, because amines in combination with nitrosating agents produce carcinogenic nitrosamines, their use in cosmetics should be limited to the lowest levels technically possible [90]. Chemical reactions of secondary amines, including ethanolamine, DEA, and TEA, with sodium nitrate (III) under various factors, such as pH, temperature, fluorescent, ultraviolet (UV), and visible (VIS) light, can produce NDELA, which is potentially carcinogenic to humans. Therefore, to reduce the concentration of NDELA, it is recommended to store cosmetics under refrigerated conditions and to add nitrosation inhibitors—vitamin E or vitamin C in concentrations of 100 to 1000 µg/mL to cosmetic preparations [91].

#### 4.1.2. Formaldehyde and Its Donors

Formaldehyde (formic aldehyde, methanal, HCHO) is the simplest chemical compound containing a carbonyl (aldehyde) group. It is a colorless gas with a characteristic odor. It is most often found in an aqueous solution called formalin. In cosmetic products, formaldehyde acts as a preservative by reacting with bacterial proteins and interfering with the function of the vital processes of these pathogens [92]. Formaldehyde donors—formaldehyde-releasing substances—are chemical compounds that, in the presence of water, can be a source of aldehyde molecules. Formaldehyde donors that commonly occur in cosmetics are shown in Table 4 [8]. The listed compounds are used as preservatives. In an aqueous environment, they release free formaldehyde, a process dependent on the type of compound, its concentration, water content, and pH of the cosmetic. The formaldehyde formed dissolves in sweat droplets on the skin’s surface. Formaldehyde’s release increases as the temperature of the cosmetic product increases and as the product is stored for a longer period of time. Formaldehyde accumulation then occurs, thus increasing exposure [33,92].

Regulation 1223/2009/EC of the European Parliament and the Council of the European Union classifies formaldehyde as a substance that can only be contained in nail-care preparations (Restrictions and Limitations, Annex III/13). Cosmetics intended for makeup may use its donors, which the aforementioned regulation classifies within Annex V, the list of preservatives allowed in cosmetic products with their permitted maximum concentrations [8,15]. Quaternium (Table 1—Item 42), DMDM hydantoin (Table 1—Items 8 and 15), imidazolidinyl urea (Table 1—Items 8, 11, 24, 44, and 46), and diazolidinyl urea (Table 1—Item 29) were identified in the analyzed cosmetics.

The potential carcinogenic effect of formaldehyde is the subject of ongoing scientific research, the multiplicity and contradictory results of which prevent conclusive confirmation of its carcinogenic effect. However, its possible pro-cancer mechanism, involving the induction of the formation of stable cross-links between the nitrogenous bases of DNA nucleotides, causing damage to the DNA, and the possibility of creating mutations that predispose to cancer, has been recognized. Studies by Rager [31] showed the effect of formaldehyde on the expression level of miRNA, which is responsible for regulating gene expression. The study observed reduced miRNA expression within formaldehyde-treated cells. A similar relationship is observed within many types of cancer [31,32].

Experimental studies conducted between 2013 and 2019 have shown the toxic effects of formaldehyde on various organs, such as the lungs, upper respiratory tract, bone marrow, and brain, as well as on cells [93].

There are reports in the literature indicating the possibility of nasal tumor formation in response to formaldehyde exposure. Key in this aspect, however, is the time of exposure, the high concentration, and the route of its administration. The results of the cited 2004 study by Hauptmann et al. primarily relate to occupational exposures via inhalation [94]. There are also speculations about inhalation exposure to formaldehyde evaporating from applied cosmetic products. On the other hand, there are no reports in the literature specifying inhaled doses of formaldehyde and/or its concentrations, so it can be concluded that exposure is marginal with short-term contact. However, it is worth noting the possibility of harmful effects of formaldehyde in the context of long-term exposure in the case of cosmetic products used for makeup, which, despite their low content, can deliver it to the skin for many years [95]. Although the mechanism of formaldehyde absorption through the skin is not yet fully understood, available results from laboratory animal studies indicate high absorption of formaldehyde from the skin surface during the use of cosmetic products containing both free formaldehyde and its donors as a preservative [32]. With regard to formaldehyde donors, available research results refer only to released HCHO, but nevertheless, 2-bromo-2-nitropropane-1,3-diol (bronopol) has been specified, which, in combination with nitrogen donors, forms potentially carcinogenic nitrosamines [32,33].

Based on current scientific reports, the IARC has classified formaldehyde within Group 1—substances with proven carcinogenic effects [8,96].

Lopez-Sanchez et al. evaluated the effect of formulation composition on the dermal absorption (in an in vitro porcine ear model) of three preservatives, namely bronopol, bronidox, and formaldehyde, as well as the absorption of formaldehyde from bronopol and dimethyldimethyl hydantoin (DMDM hydantoin). The aqueous solution, oil/water (o/w) emulsion, and hydrogel were tested. The absorption of bronidox and bronopol was shown to depend on the formulation. In the case of the transdermal absorption of formaldehyde released from bronopol, it also depends on the formulation. The degree of transdermal absorption of all preservatives tested is low, so regulatory concentrations can be safely used [33].

#### 4.1.3. Parabens

Parabens (or nipagins) are esters of 4-hydroxybenzoic acid (Table 5). They are colorless and odorless crystalline solids which act as preservatives in cosmetic products and exhibit a broad spectrum of antimicrobial activity. They are commonly used in cosmetics due to their lack of effect on cosmetic properties, such as taste, smell, color, and texture [97,98]. Parabens were found in 28 cosmetics of the analyzed 50 (Table 1, Items 3, 5–7, 9–12, 15, 19, 21, 23, 25–34, 37, 41, 42, 44, 48, and 49).

According to Regulation 1223/2009/EC of the European Parliament and of the Council of the European Union, methylparaben, ethylparaben, propylparaben, and butylparaben are included in the list of preservatives permitted in cosmetic products (Annex V/12 of Regulation 1223/2009/EC). However, concentration standards have been set for them, specifying that their content is a maximum of 0.4% by weight of a single paraben and 0.8% by weight of a mixture of parabens in a cosmetic preparation. Isopropylparaben, isobutylparaben, and benzylparaben are banned substances for use in cosmetics (Annex II, Regulation No. 1223/2009/EC of the European Parliament and the Council of the European Union). They were withdrawn from use in 2014 by Commission Regulation 358/2014 [8,99].

Parabens have been the subject of numerous studies. Pedersen et al. conducted studies on the human isolated epidermis and demonstrated the ability of parabens to penetrate transdermally, with the level of absorption depending on the structure (length of the alkyl substituent) of the tested compound. The penetration ability was ranked as methylparaben < ethylparaben < propylparaben < butylparaben and increased with the application of an occlusive dressing. After penetrating the skin barrier, parabens enter the bloodstream, where they are transported to the relevant organs. Most of them are metabolized; however, they can also accumulate in the adipose tissue [34].

Esters of *p*-hydroxybenzoic acid can bind with estrogen receptors to exhibit properties of xenoestrogens. Once attached to the receptors, mimicking the action of estrogen, they stimulate cell growth and affect genes controlled by the aforementioned receptors. Okubo et al. studied the estrogenic activity of parabens and showed that it is much weaker than that of natural estrogen. Many opinions on the safety of using parabens as preservatives in cosmetics are based on these studies [35]. However, they are also used as drug and food additives. Thus, the daily dose delivered to the body may be much higher than expected and sufficient to equal or exceed the estrogen activity recommended [97,98]. Oishi’s study of male rats that were orally administered propylparaben found a decrease in serum testosterone levels, as well as a decrease in sperm count. This suggests an adverse effect of parabens on the male reproductive system [100]. Harmful effects on the female reproductive system were demonstrated in a Korean study, which confirmed an increase in uterine weight in laboratory animals following the administration of parabens [36].

Due to the xenoestrogen properties of parabens, there are reports of their effects on breast cancer formation. A 2004 study by Darbre et al. found an increased risk of breast cancer in women using antiperspirants containing 4-hydroxybenzoic acid esters. Parabens have also been detected in human mammary gland tumor tissue [37]. Barr et al. also found the presence of parabens in mammary gland tissue in female subjects. Some of the subjects, however, reported no antiperspirant use. Nipagins must therefore have come from other sources [101].

The effects of parabens on the skin and the possibility of stimulating skin cancer were studied by Handa et al. The authors of the study [102] showed that methylparaben exposed to ultraviolet B (UVB) radiation induces oxidative stress and the oxidation of keratinocyte lipids. This can cause skin damage, including damage to the genetic material of skin cells, leading to the development of cancer.

The IARC does not provide a classification for *p*-hydroxybenzoic acid esters. According to a 2010 SCCS report, parabens used in cosmetic products are completely safe and have no toxic or carcinogenic effects. The only restriction on their use is that they are kept to concentrations not exceeding 0.1% [97,98].

The toxicity of parabens has been demonstrated in animal and in vitro studies; however, the data cannot be considered fully reliable due to the unlikely exposure/safety profile [97]. Concerns include their effects on endocrine activity, carcinogenesis, infertility, spermatogenesis, adipogenesis, perinatal exposure, and non-allergic skin, as well as the psychological, and ecological effects [38]. Several studies have shown parabens to be non-teratogenic, non-mutagenic, and non-carcinogenic, and actual evidence of their toxicity in humans has not been established. It is currently believed that methyl, ethyl, and propyl parabens are safe for use in cosmetics and pharmaceutical products within the recommended dose range [97]. It can be concluded that parabens as preservatives in cosmetic products have convincing data supporting the absence of significant dermal toxicity [38].

#### 4.1.4. Tert-Butyl Compounds

*Tert*-butyl-substituted compounds used in cosmetics include *tert*-butylated hydroxyanisole (BHA) and *tert*-butylated hydroxytoluene (BHT, Table 6). These substances are used in cosmetic preparations for their preservative and antioxidant properties. BHA is a waxy whitish-colored substance. According to INCI nomenclature, it is designated as BHA, Antioxyne A, Antrancine 12, Embanox, and Tenox BHA. Meanwhile, BHT occurs in the form of whitish crystals and, according to INCI, is written as BHT, *tert*-butylated hydroxytoluene, Agidol, Antrancine 8, and BP alcophene [8,39,40]. BHT was found in 14 analyzed cosmetics (see Table 1—Items 5, 6, 14, 23, 28, 32, 37, 38, 44–47, and 50).

BHA and BHT have been extensively studied in regard to their safety for many years. This is supported by the fact that these substances are widely used, both in cosmetics and food. However, their effects on the human body have not been fully confirmed and are unclear. Studies point to their anticancer, as well as pro-cancer, properties. The anticancer mechanism, according to available analyses by Saito et al., may involve the induction of cytochrome P450 and enzymes (including glutathione transferase) by *tert*-butyl compounds that catalyze the detoxification of xenobiotics, whose products are carcinogenic. In addition, their antioxidant properties can neutralize free radicals that can cause damage to the DNA double helix, leading to mutations [39].

In opposition, there are reports of pro-oxidant activity. Although in vitro studies by Sablin et al. indicate the ability to inactivate the p53 protein or inhibit TP53 (the gene encoding the p53 protein) transcription at high doses of BHA, the underlying mechanism has yet to be fully elucidated and requires further study. However, the effects of p53 protein inactivation are well-known, consisting of redox disruption, DNA oxidation, and an increase in mutation rates leading to genetic disorders, excessive cell proliferation, and tumor formation [103]. The cellular toxicity of *tert*-butyl hydroxyanisole, specifically the induction of apoptosis, has also been reported. In a study by Yu et al., BHA was incubated with isolated cellular mitochondria and resulted in the release of cytochrome c and activation of caspases leading to apoptosis [41]. The possible influence of *tert*-butylated compounds on endocrine disruption has also been indicated. Pop et al., based on in vitro tests, inferred that *tert*-butyl compounds, especially BHA, have weak estrogenic effects. Among other things, they studied the proliferative effects on human breast cancer cells, and *tert*-butylated hydroxyanisole showed an affinity for estrogen receptors [42].

The Directorate General for Health and Food Safety (SANTE) recognizes the strong evidence supporting the potential for *tert*-butyl hydroxyanisole to cause endocrine disruption in humans. Its carcinogenic potential, on the other hand, was assessed by the IARC as possibly occurring and is classified as a Category 2B carcinogen due to the development of gastric tumors in rodents following chronic exposure to high dietary concentrations [43]. In contrast, BHT belongs to Category 3 according to the IARC classification and is not considered a human carcinogen [15,42]. It is worth mentioning that there is widespread concern about BHA among consumers, stemming from the belief that it is a carcinogen. Although many regulatory agencies have established safe exposure limits for BHA, IARC’s classification and Proposition 65′s listing resulted in the addition of BHA to the list of banned substances in children’s food products, as well as mandatory reporting of its presence on product labels. The classification of a substance into a specific group often precludes the possibility of conducting an exposure-based risk assessment [43].

Baran et al. attempted to evaluate the potential mechanism of BHA-induced toxicity on the molecular level in zebrafish embryos (*Danio rerio*). For this purpose, zebrafish embryos were exposed to BHA at concentrations of 0.5, 1, 5, 7.5, and 10 ppm and then evaluated after 24, 48, 72, and 96 h. The survival rate, hatching rate, and rate of developmental defects were evaluated. The apoptosis and histopathological characteristics of zebrafish larvae exposed to *tert*-butylhydroquinone (TBHQ), one of the main metabolites of BHA, at doses of 0.5, 2.5, 3.75, and 5 ppm were also examined. ROS were found to be responsible for the changes produced in embryos. It has been indicated that the induction of ROS formation, occurring during exposure to BHA and/or multiple hydroxyl groups, may be responsible for apoptosis [104].

Mizobuchi et al. showed that BHA induces apoptosis, while BHT induces non-apoptotic cell death in rat thymocytes. These results confirm the safety of BHA and indicate the importance of assessing toxicity not only at the tissue level but also at the cellular level [105].

For BHA and BHT, the CIR expert panel has set the maximum concentration limits for these substances at 0.5% due to their uncertain toxicological profile and potential irritant effects on the skin and mucous membranes [106].

#### 4.1.5. Ethoxylated Compounds

Ethoxylation is a chemical reaction involving the introduction of ethoxyl groups into alcohols or phenols, using ethylene oxide (EtO) [107]. Ethoxylated compounds used in cosmetics include polyethylene glycols (ethylene glycol polymers, abbreviated as PEGs). PEG, or poly(EtO), is a polymer obtained mainly by anionic polymerization, which, depending on the chain length (resulting from the number of moles of EtO used), is a water-soluble solid or liquid. In cosmetic products, PEGs act as emulsifiers, solvents, and substances that improve the viscosity of the finished cosmetic product. In INCI nomenclature, they appear under the term “PEG” with a distinguishing numeral indicating the number of moles of EtO and the substance ethoxylated (Table 7). The next compounds are ethoxylated fatty alcohols. These include laureth—ethoxylated lauryl alcohol; steareth—ethoxylated stearin alcohol; ceteth—ethoxylated cetyl alcohol; and ceteareth—an ethoxylated mixture of cetyl and stearin alcohols. In cosmetics, they perform functions identical to PEGs but also act as foaming agents and surfactants [8,107]. These compounds were found in tested cosmetics (laureth derivatives—Table 1, Items 14, 18, 20, 46, and 50; steareth derivatives—Items 15, 16, and 43; and cetyl derivatives—Items 14, 15, 17, 32, and 47–49).

Ethoxylated compounds are allowed to be used in cosmetic products without quantitative restrictions, except for several representatives of this group which are included in Annex III of the Regulation of the European Parliament and of the Council of the European Union No. 1223/2009/EC, for which concentration restrictions have been designated [8]. Ethoxylated ingredients are considered safe for cosmetic use based on the opinion of the CIR (an organization that studies the safety of cosmetic ingredients) and studies conducted in Germany and Korea. Noteworthy, however, is the purity of ethoxylated compounds. Particularly dangerous contaminants resulting from the formation of 1,4-dioxane, ethylene glycol (EG), and residues of unreacted EtO exhibit potential toxic effects, are a potent poison or have confirmed carcinogenic effects, respectively [107].

1,4-Dioxane can penetrate the skin and cause skin inflammation, which can lead to tumorigenesis [44,45]. Epidemiological studies of human exposure published by the NTP in 2014, indicated the occurrence of pulmonary edema, lung damage, and kidney and liver tumors leading to death after exposure to 1,4-dioxane vapors [46]. However, there is insufficient evidence supporting its carcinogenicity in humans. In 2001, lung inflammation and histological changes involving the liver and kidneys were observed in experimental animals tested in New York after exposure by inhalation to 1,4-dioxane. Laboratory rats that inhaled appropriate concentrations of 1,4-dioxane showed an increased incidence of liver tumors [46]. The effect of 1,4-dioxane on the occurrence of morphological changes in the kidneys and liver of rats, including changes in liver enzymes, was also demonstrated. However, the mechanism of its potential carcinogenicity has yet to be fully understood. A possible effect of 1,4-dioxane is on DNA biosynthesis. The compound passes into the cell nucleus, where it binds to DNA. This binding is thought to interfere with RNA transcription processes through the effect of 1,4-dioxane on the activity of ribonucleic acid polymerases, as was observed in studies on rat liver [47,48].

EtO (epoxide, oxirane) shows some pro-cancerous properties. There is evidence of an association between EtO exposure and the occurrence of cancer in humans. Epidemiological studies by Steenland et al. and Wong et al. conducted on workers in industrial plants where EtO was used as a gaseous sterilizing agent demonstrated a slightly increased incidence of lymphatic and hematopoietic cancers, particularly in men. Women with occupational contact with EtO sterilizers showed an increased risk of breast cancer [49,50]. Inhalation exposure in laboratory animal studies, according to the NTP, caused tumors in the hematopoietic system and uterus of mice [46]. The mechanism of carcinogenicity is considered in terms of EtO’s genotoxic and mutagenic properties. It is believed that the reaction of EtO with DNA initiates a cascade of genetic events that lead to cancer. This alkoxylating compound can form DNA adducts, leading to genetic mutations. EtO also causes chromosomal aberrations, particularly deletions, which can cause genetic changes and lead to cancer [108]. The IARC and the United States Environmental Protection Agency (USEPA) have classified EtO as a known human carcinogen. Both agencies were adamant that epidemiological evidence for lymphatic and breast cancers was “limited”, but that evidence from animal studies was “sufficient” and “extensive” (respectively) that EtO is genotoxic [51]. Epidemiological studies have not shown an increased risk of breast cancer or lymphohematopoietic malignancies (LHMs). Similarly, toxicology and biomarker studies in animals and humans have not provided strong indications that EtO causes LHM or breast cancer. Ultimately, animal data are insufficient to determine the true shape of the dose–response relationship or predict with any certainty the tumor response at very low doses [51].

The IARC classifies 1,4-dioxane as potentially carcinogenic, i.e., Category 2B, while EtO is classified as a compound with proven carcinogenicity, i.e., Category 1 [15]. Regulation 1223/2009/EC of the European Parliament and the Council of the European Union prohibits the use of 1,4-dioxane and EtO as ingredients in cosmetics [8]. However, these compounds can be contaminants of cosmetic products as a result of technological processes. At the same time, the presence of these impurities does not have to be listed on cosmetic labels [8,109]. The Scientific Committee on Consumer Safety conducted a safety assessment of dioxane contaminants in cosmetic products in 2015 which concluded that the concentration of these contaminants does not exceed the dangerous values for human health. However, these analyses do not take into account the effects of long-term low-dose exposures to this compound [110].

### 4.2. Potential Carcinogenic Effects of Black Carbon and Silica in Cosmetics?

#### 4.2.1. Carbon Black

Carbon black (charcoal) is a black-colored substance produced from wood through a dry distillation process. It is produced in a fine, light powder that is used in cosmetic products as a pigment, giving a cosmetic a dark or black color [111]. The Colour Index International (CI) gives carbon black a number of CI 77266 [75]. Carbon black was found in the analyzed cosmetics (Table 1—Items 1, 2, 10, 12, 13, 39, 40, and 41).

According to Regulation No. 1223/2009/EC, carbon black was included in Annex IV —the list of “colorants permitted in cosmetic products” (IV/126). Annotation of the required purity was also added, which refers to the maximum possible concentrations of contaminants such as arsenic, lead, mercury, and polycyclic aromatic hydrocarbons [8]. The possibility of harmful effects of carbon black through the presence of polycyclic aromatic hydrocarbons was considered. However, this thesis has been refuted, as hydrocarbons can only be extracted with certain solvents and high temperatures, making the process impossible on the skin surface [76,112]. Studies were also conducted on animals (rats) that inhaled air-suspended carbon black [77]. Based on the results, the potential possibility of mutations in the genes of alveolar epithelial cells was identified. Importantly, the occurrence of these changes is thought to be related to the occurrence of chronic inflammation as a result of “lung overload” [77]. Regular inhalation of carbon black may contribute to reduced lung functioning in humans, as confirmed by epidemiological studies by Puntoni [112] and Wellmann [78]. According to the available literature, when used long term, the carbon black in makeup cosmetics applied around the eyes (mascara and pencils) causes reddening of the eyelid conjunctiva. A study published in an IARC monograph that was conducted on mice receiving lifelong applications of carbon black in acetone extracts showed an increased incidence of cutaneous squamous cell carcinomas and dermal papillomas. However, similar studies conducted using monkeys showed changes in epidermal thickness (including atrophy) and skin fibrosis. Dry carbon black powder applied to the skin for 24 months did not cause skin tumors in any of the animals studied [76,79,111]. Epidemiological studies involving workers having occupational contact with carbon black by inhalation and dermal routes had an increased incidence of excessive dermal keratosis, as well as leukoplakia. These lesions represent known conditions that can predispose to the formation of cancerous foci, so-called precancerous conditions [79,80,113].

The IARC, on the basis of animal experiments, has classified carbon black as a probable human carcinogen—Group 2B [111].

#### 4.2.2. Silica

Silica (silicon dioxide) is a compound commonly found on Earth. As a mineral, it forms, among other things, quartz. In cosmetic products, it is found in the form of colloidal particles or gels. Silica acts as a filler, regulating the density, viscosity, and flowability of powders. It also absorbs moisture from the skin surface, exhibits an abrasive function in toothpaste, and improves the adhesion and spreadability properties of cosmetic products. According to the INCI, silica is described as silica or silicon dioxide, while silica gel is described as hydrated silica [8,81]. Silica was found in 24 of the 50 analyzed cosmetics.

Silicon dioxide is a substance permitted for use in cosmetics, with no quantitative restrictions. Moreover, it is used in ointments and medicinal creams as an auxiliary ingredient that imparts certain properties to the preparation. Silica provides the skin with silicon, which participates in the formation of collagen in the body, supports the skin’s repair processes, regulates sebum secretion by sebaceous glands, and affects the sealing of blood vessel walls. Silicon is therefore an essential element for the proper functioning of the body. However, according to modern scientific research, silica is not a completely inert substance for the body [8,81,114].

Silica used in cosmetics or to form colloidal particles or gel can be in the amorphous or crystalline form. In terms of potential harm, the crystalline, non-hydrated form is more often mentioned. It has been proven that long-term inhalation of airborne crystalline silica dust causes pneumoconiosis, vascular disease, and silicosis of the lungs caused by multiple fibrosis. These conclusions were based on the results of an epidemiological study by Brown and Rushton [82] that was conducted on workers occupationally exposed to silica dust. Extensive laboratory studies were conducted in South Korea, using silicon dioxide micronized into nanoparticles (particles in the range of 1–100 nanometers). It has been theorized that the reduction in silica particle size would allow them to be more easily accessible to the body through various penetration routes [83]. Considering that the skin is the largest organ of the body, it is inferred that the probability of absorption of nanoparticles is high. It is also worth noting that, despite the recognition of silica macroparticles as a safe substance, their micronized form may have harmful properties. Chang’s in vitro studies reported a cytotoxic effect by inducing the production of ROS that damages cellular DNA [84]. Lin et al. suggested the sensitivity of lung cells exposed to silicon dioxide due to oxidative stress. In addition, it has been confirmed that the prolonged presence of silica in the lungs causes activation of macrophages; prolonged release of pro-inflammatory cytokines, resulting in the appearance of inflammation; and stimulation of neutrophils to produce free radicals that, in turn, induces genotoxicity, resulting in the appearance of neoplastic lesions [85]. Lin et al. reported that silica nanoparticles can cross the skin barrier and localize in lymph nodes [85]. This is contradicted by the results published by Hirai et al. [115]. These findings suggest the need for additional studies to analyze the dermal penetration of silica dioxide nanoparticles and the biological effects after exposure. This is due to the presence of conflicting studies refuting the previously mentioned theories [115].

IARC considers studies indicating the cellular toxicity of silica particles and the possibility of initiating intracellular oxidative stress to be sufficient to classify crystalline forms of silicon dioxide into Group 1—human carcinogens. Silica in amorphous forms, according to the IARC, has been classified as a Group 3 predisposing substance [81,116]. However, the European Union’s Scientific Committee on Consumer Safety (SCCS) issued an opinion stating that there was insufficient evidence indicating the harmful effects of silicon dioxide nanoparticles in all forms, deeming studies incomplete. At the same time, the opinion stressed that there is no evidence establishing the complete safety of silica-containing formulations [116]. However, there is risk of inhalation of silica-containing powder particles during the application of makeup. Therefore, the risk of this type of exposure exists and should be considered.

### 4.3. Contamination with Selected Heavy Metals

Heavy metals are referred to as a collection of metals and semi-metals characterized by a high density (above 4.5 g/cm^3^) and often toxic properties [117].

The reactivity of heavy metals is due to the participation of electrons from d-type orbitals in reactions. This, in turn, enables them to form complexes with organic ligands, as well as affect their catalytic properties. Transition metal compounds can initiate a number of biochemical reactions. In trace amounts, they are used for the normal functioning of the body. However, when introduced in higher concentrations, they exhibit toxic properties, including carcinogenicity.

The absorption of metals from cosmetics through the skin is low [118]. However, due to the proven ability of some of them to accumulate in the human body, even a small concentration of metals in a cosmetic used over several years can result in the accumulation of these elements in the skin and internal organs, leading to toxic effects. There are also known cases of allergic dermatitis and systemic effects due to metals contained in cosmetics [119].

Compounds of some metals, as authorized by European Union law, are used in the cosmetics industry mainly as UV filters (e.g., TiO_2_ and ZnO) and pigments in color cosmetics (e.g., Ag, Au, Bi_2_O_3_, CaSO_4_, AgNO_3_, Cr_2_O_3_, Cr(OH)_3_, Co_2_O_3_, FeO, Fe_2_O_3_, etc.).

Due to the confirmed or potential carcinogenic properties of many heavy metals (e.g., Cr, Cd, Ba, and Pb), their intentional addition to cosmetic products is prohibited. Contamination of a cosmetic preparation with them can occur as a result of the manufacturing process or improper purification of naturally derived raw materials (e.g., mineral oils, paraffin, silicones, and aliphatic hydrocarbons) used in cosmetics. European Union regulations do not specify safe levels for concentrations of technological contaminants in cosmetics and allow trace amounts of prohibited heavy metals provided that the final formulation is safe for human consumption [8,99]. However, the literature indicates that it is possible to contaminate cosmetics with heavy metals such as arsenic, cadmium, lead, and mercury. Therefore, their toxic effects are described below [22,23,24,25].

Most heavy metals are considered highly toxic. There are multiple routes of exposure, including ingestion, inhalation, and dermal absorption, that can result in certain health effects. It has been noted that the effects of heavy metals on children’s health are more severe than in adults. The harmful effects of these elements on children’s health include mental retardation, neurocognitive disorders, behavioral disorders, respiratory problems, cancer, and cardiovascular disease [120].

European Union legislation does not regulate in detail the maximum permissible concentrations of heavy metals in cosmetics. However, every cosmetic approved for marketing is subject to a safety assessment, which includes an evaluation of the degree of heavy-metal contamination. A single application of a cosmetic product that contains toxic elements does not pose a health risk, as the concentrations are usually low. However, long-term use of contaminated cosmetics, especially those applied to certain areas (e.g., lips and eyelids) from which the elements can easily penetrate the body and accumulate, can result in lesions.

The degree of exposure to heavy metals depends on the occurrence and concentration in cosmetics, as well as the amount of cosmetic product used, exposure time, application site, and frequency of use [57,121].

Due to the sourcing and manufacturing process, dyes are considered to be the ingredients with the highest content of metallic impurities, and the degree of cosmetic contamination increases proportionally with their usage. The presence of metallic impurities is not usually highlighted on the labels of cosmetic products, so by reading only the ingredients of the cosmetics, it is not possible to obtain information about the impurities [57,121].

The most undesirable and dangerous metal contaminants include elements such as arsenic, cadmium, lead, and mercury [10,57,118].

Lim et al. suggest that heavy metals present in cosmetics do not appear to pose a serious health risk. However, for those who frequently use oral cosmetics, contamination with certain heavy metals, such as lead, manganese, and chromium, should be minimized [25].

Contamination studies were carried out by Saadatzadeh et al. The data obtained indicated that the lead content of the studied products did not exceed the permissible limit set by the Federal Office for Consumer Protection and Food Safety in Germany (BVL). Additionally, cadmium values in all products were well below the limit set by the BVL. The arsenic content of lipsticks, eyeshadows, and eyebrow pencils was much higher than BVL standards, while the mercury content was much lower than BVL standards. Commercially available cosmetics fared better, with the exception of mascara, which had a higher arsenic content than cosmetics from outside the official market. The high arsenic content in eyeshadows and eyebrow pencils from unknown sources is an issue that should be taken into account by the relevant authorities [24]. Moreover, Arshad et al. studied the content of heavy metals, including cadmium, chromium, iron, nickel, and lead, in various brands of lotions, foundations, whitening creams, lipsticks, hair dyes, and sunscreens, using atomic absorption spectrometry. They noted that they had the highest concentrations of nickel, lead, and chromium (7.99 ± 0.36, 6.37 ± 0.05, and 0.43 ± 0.01 mg/kg, respectively), while lipsticks had only elevated iron levels (12.0 ± 1.8 mg/kg). In contrast, cadmium levels were highest in lotions (0.26 ± 0.02 mg/kg). The regular use of these products can cause serious human health risks, especially skin cancer, with long-term exposure [23]. A study from Pakistan found high concentrations of lead and arsenic in lipstick and eyeshadow samples. Concentrations of lead and cadmium in samples of creams and foundations were within safe levels. Most of the cosmetic samples contained heavy metals above safe levels, posing a health risk to female consumers who use them for long periods of time [122].

#### 4.3.1. Arsenic

Scientific evidence currently links arsenic to the onset of health problems. It has been proven to accumulate in keratin-rich tissues, such as the skin, nails, and hair. Dermal exposure causes a variety of skin lesions. Prolonged exposure stimulates melanocytes to produce melanin, which manifests as the formation of skin hyperpigmentation with foci of hyperkeratosis. However, in addition to the possibility of causing cosmetic defects, the possibility of toxic effects of arsenic is assumed. An association between arsenic exposure and the occurrence of precancerous conditions and skin cancers, including Bowen’s disease and basal cell carcinoma, has been documented [52,56].

A 2011 study of 49 makeup products in Canada found that arsenic contamination concentrations exceeded acceptable limits in two of the cosmetics tested. These were lip-tinting products exceeding the designated arsenic concentration standards by several times. The standard was set at 3 μg/g body weight per day, while the cosmetics in question showed arsenic contamination at levels of 70 μg/g and 12 μg/g [22,25].

There is also a link between arsenic and lung cancer. The mechanism leading to cancer may involve arsenic acting at the level of tumor promotion by modulating signaling pathways responsible for cell growth. In particular, increased transcription levels of genes for keratinocyte growth factor have been observed, contributing to their uncontrolled proliferation and resulting in tumorigenesis of skin cancers. The mechanism of arsenic’s carcinogenic effect may be due to the formation of ROS within the cell, leading to oxidative stress. This, in turn, promotes damage within the DNA double helix, leading to genetic mutations and, consequently, cancer [53].

Arsenic is currently considered a tumor agent in breast, lung, and other organ cancers [54,55]. The IARC classifies arsenic in Group 1, substances with proven carcinogenic effects, based on sufficient evidence of carcinogenicity to humans [15].

#### 4.3.2. Cadmium

Cadmium and its compounds enter the body mainly by inhalation. However, they also can penetrate through the skin. After getting into the body, they enter the bloodstream, and from there, they are transported mainly to the liver and kidneys. These are the target organs of the metal’s toxic effect. Despite the harmful effects of cadmium, poisoning by this element is rare and only occurs during long-term inhalation exposure. Symptoms of poisoning include fever, pneumonia, respiratory failure, yellow discoloration of the roots of teeth, and liver and kidney dysfunction [57].

According to a study by Gondal [123] on lip-staining products, cadmium concentrations ranged from 4.9 to 10.6 μg/g. A study conducted in New Zealand on 557 lipsticks found cadmium contamination levels between 1.1 and 3390 μg/g. A Canadian study in 2011 on 49 different makeup cosmetics found cadmium in 25 cosmetics, with an average value of 0.3 μg/g, which is below Canada’s established standard of 3 μg/g [25].

However, there is a risk of inhaling powder or loose cosmetics particles while applying makeup. Consequently, the risk of this type of exposure exists and should be considered. Exposure to cadmium is well described in the literature. An epidemiological study by Sorahan et al. on workers of cadmium-using factories with inhalation exposures showed an association between exposure to the metal and the occurrence of lung cancer and prostate cancer [58]. Waalkes, Il’yasova, and Schwartz found a correlation between cadmium exposure and an increased incidence of lung, prostate, and kidney cancers [59,60]. In similar studies conducted by Goyer on laboratory rats exposed to cadmium by inhalation and ingestion, the same results were obtained. The formation of testicular, prostate, and kidney tumors in animal studies was confirmed [124]. Another evaluation of cadmium’s carcinogenic potential was conducted by McElroy et al., who found an increase in breast cancer in women [61]. Cadmium is also thought to cause cancers of the stomach and breast and other internal organs [125,126].

Studies have pointed to a potential mechanism for cadmium’s carcinogenic effects, which may involve oxidative stress and decreased antioxidant potential. Excessive amounts of ROS, with weakened antioxidant mechanisms, promote the activation of protooncogenes, and this, in turn, leads to the overproduction of proliferation-stimulating factors, tissue hypertrophy, and the possibility of tumor formation. As previously mentioned, cadmium shows the ability to impair the action of enzymes, including those involved in DNA repair and removal of damaged DNA, which was confirmed by experimental studies [62]. Waisberg’s research [63] indicated that cadmium is capable of disrupting intercellular signaling, which is important for cell growth and differentiation. According to Waisberg, the element modifies specific signaling molecules (*β*-catenins) capable of binding to transcription factors and leads to changes in gene expression. The effects of the described element on the operation of cellular signaling pathways result in a disruption of the reception and processing of external signals within the cell, which prevents the proper functioning of cells and can lead to cancerous transformation. *β*-catenins are also responsible for cell adhesion. Abnormal adhesion is a known factor responsible for cancer progression [63]. On the other hand, according to a study by Poirier and Vlasov, cadmium inhibits DNA methylation. DNA methylation regulates the expression of genes, including those responsible for cell division, growth, and differentiation. The hypomethylation of genes caused by cadmium results in the excessive synthesis of factors responsible for cell proliferation, leading to the development of cancerous tumors [64].

Based on existing studies, a close link between cadmium exposure and the occurrence of human cancers exists. The IARC has placed cadmium and its compounds in Group 1—substances with proven carcinogenic effects [15].

#### 4.3.3. Lead

Lead, as one of the metals that are contaminants of cosmetic ingredients, shows the ability to penetrate the skin. After penetrating the skin and being transported through the bloodstream, it accumulates in tissues and organs. Particularly vulnerable to its effects are the kidneys and the brain. Currently, there is a presumption that there is no threshold level for the toxic effects of lead, and even exposure to low concentrations of lead can have adverse effects, especially on young children. Lead poisoning manifests as disorders of the nervous system, including difficulty concentrating, delayed reaction times, and headaches. In addition, anemia and abdominal pain are commonly seen [57].

A 2011 study of 49 makeup cosmetics in Canada found low concentrations of lead in the products tested, except for lip-tinting products. In these products, the lead standard considered safe (10 μg/g body weight per day) was exceeded by several times (110 μg/g in lip gloss and 28 μg/g in lipstick). In a study from New Zealand evaluating 557 lipsticks, 35 products exceeded the test metal standards. In addition to lipstick products, lead contamination of other facial cosmetics was negligible; however, Silbergeld et al. reported that these cosmetics may not be fully safe [25,127]. Importantly, Silbergeld found that the concentrations of lead needed to cause carcinogenic effects are lower than the concentrations generally considered toxic to humans [15,127].

Epidemiological studies have pointed to the nephrotoxic effects of lead, which can lead to kidney tumors in humans. A similar study of the toxic effects of lead on the kidneys was conducted on laboratory animals, in which an increase in the incidence of kidney tumors was observed with exposure to high doses of lead. An increase in the incidence of lung and gastric cancers was found among pigment plant workers exposed to the inhalation of dust containing lead and its compounds. A correlation between the level of cellular DNA damage and the length of service with lead exposure was observed. The effect of lead on inflammatory reactions has also been recognized. Among other things, there is an induction of endothelial cells and Langerhans cells to produce pro-inflammatory cytokines. Long-term inflammatory processes in the body may predispose to carcinogenesis. Lead also enhances the formation of free radicals that damage cellular structures, including many enzymes and nucleic acids. The activity of glutathione is reduced, resulting in an imbalance between the formation of free radicals and the production of antioxidants. As a result, an impairment of DNA repair mechanisms occurs, and this can lead to the abnormal uptake of genetic errors. However, the lack of sufficient evidence means that the mechanism of lead’s potential carcinogenicity is not clear and requires further research [65,66,67].

The IARC recognizes lead and its compounds as a probable human carcinogen—Group 2A [15].

#### 4.3.4. Mercury

Mercury, as well as its derivatives, has strong adsorption through the skin and its appendages, namely hair follicles, sweat glands, and sebaceous glands. Mercury derivatives used in cosmetics include thimerosal (merthiolate, vitaseptol). According to the INCI, thimerosal and phenylmercury salts, phenyl mercuric acetate, and phenyl mercuric benzoate act as preservatives. These derivatives are listed in the Regulation of the European Parliament and the Council of the European Union No. 1223/2009/EC as preservatives permitted for use in cosmetic products with a maximum concentration of mercury of 0.007% by weight (Annex V/16 and V/17) [8,57].

The determination of mercury contamination in makeup cosmetics has shown low values of mercury that are within accepted standards. The exceptions were products intended for application to the eye area—mascaras, eye pencils, and eyeshadows—where high values of mercury concentrations were recorded [25].

The toxic effects of mercury after dermal exposure primarily include the occurrence of skin inflammation and contact allergies. Dark ashy discoloration localized in the skin folds resulting from the deposition of mercury compounds in the dermis is also characteristic. Mercury poisoning is manifested by the occurrence of disorders of the gastrointestinal and nervous systems. Data on the carcinogenic effects of mercury on humans are insufficient and inconclusive to classify mercury as a confirmed carcinogen. There are epidemiological studies reporting increased mortality among workers exposed to mercury vapor [68,69,70,71]. However, the results regarding the relationship between mercury exposure and an increase in cancers (lung cancers) have been contradictory [72,73]. In studies using laboratory animals, mercury was found to have damaging effects, but no tumors were observed. In contrast, a consequent study by the NTP showed an increase in the incidence of renal tubular carcinomas in rats following intragastric administration [128]. Queiroz et al. [74,129] confirmed the mutagenic effects of mercury after testing the blood of workers occupationally exposed to mercury. They showed an increased incidence of micronuclei in cells compared to the control group. An analysis showed that the number of micronuclei determines the susceptibility to cancer, as it determines the degree of damage to the cell’s genetic material. Studies have shown that mercury causes defects in the efficiency of DNA repair mechanisms. The mechanism of mercury’s potentially carcinogenic effects is not fully understood. The pro-oxidant properties of mercury are associated with damage to DNA and increased production of pro-inflammatory cytokines [68,69,70,71,130].

Although the carcinogenic effects of mercury have not been conclusively confirmed, studies indicate possible carcinogenic effects of mercury and its compounds. The IARC found this evidence insufficient, so mercury and its compounds are classified within Group 2B [15].

## 5. Conclusions

The use of cosmetics can be associated with undesirable effects due to the presence of certain chemical substances. An analysis of makeup cosmetics available on the European market was carried out concerning substances with potential carcinogenic properties as described in recent years in the literature. Among 50 random facial makeup cosmetics, the following substances were identified as potential carcinogens: parabens (methylparaben, propylparaben, butylparaben, and ethylparaben), ethoxylated compounds (laureth-4, lautreth-7, or ethylene glycol polymers known as PEG), formaldehyde donors (imidazolidinyl urea, quaternium 15, and DMDM hydantoin), and ethanolamine, and their derivates (triethanolamine and diazolidinyl urea), as well as carbon and silica. The most common compounds with a potential carcinogenic effect in the analyzed cosmetics were parabens (28 for 50 analyzed cosmetics contained that ingredient), silica, and ethoxylated compounds. Exposure to potential carcinogens over a long period of use can be an important concern for cosmetics. Based on INCI, it cannot be concluded that any of the listed cosmetics in Table 1 may actually be absorbed sufficiently, systemically, or locally to cause carcinogenicity. Nevertheless, the makeup cosmetics are usually applied by customers for many years. Furthermore, the literature indicates that a long-term exposure to some of the substances described in this review may be potentially carcinogenic. The need for further research is well illustrated by the example of butylated hydroxytoluene (BHT) (2,6-di-*tert*-butyl-4-methylphenol), as this substance was found to promote inflammation and tumors in the lungs of male mice dosed with BHT at 150 mg/kg body weight [131]. However, it should be noted that, in a similar study, no carcinogenicity was observed when mice were fed a diet containing up to 5000 μg/g BHT for 96 weeks [132]. Therefore, the studies often have conflicting results, and further research is needed specifically related to the cosmetics market. The literature review confirmed the suppositions regarding the potential carcinogenic effects of the selected cosmetic ingredients. The relationship between the skin and makeup is noteworthy. This poses a risk of overexposure, particularly in terms of Group 2B or non-IRAC substances.

Existing regulations and the obligation to conduct safety reports on the use of cosmetic products should provide consumers with complete protection both from substances with confirmed harmful effects and those that potentially exhibit such properties. However, reports show the risk of potential contamination of cosmetics with heavy metals. Heavy metal contamination is a significant and well-known problem described in the scientific literature. This contamination needs to be carefully evaluated and taken into consideration for product launches. Cosmetics placed on the market without the appropriate permits and procedures remain a problem (cosmetics from small manufacturers, produced without authorization). Therefore, it seems necessary to carry out studies on the long-term exposure of compounds present in cosmetics and perhaps introduce stricter standards and laws regulating the potential content of heavy metals in cosmetics. This work presents the problem with long-term exposure to potentially carcinogenic substances correlated with the relation of skin with makeup cosmetics and may introduce legislation requiring confirmation of the absence of specific compounds recognized as potential carcinogens. Nevertheless, our study did not include an assessment of long-term exposure of these substances. The work should be considered as a preliminary tool for further consumer exposure assessment. Further studies should be conducted in the direction of long-term exposure.

## Figures and Tables

**Table 1:** Analysis of the INCI ingredients of selected cosmetic products for facial makeup.

No.	Type of Cosmetics *	Manufacturing Country	Potential Carcinogen (by INCI Nomenclature)	Place in INCI on Label	Sum of Potential Carcinogens
1.	Eyeshadow	Poland	CI 77266 (carbon black)	7	1
2.	Eyeshadow	Poland	CI 77266 (carbon black)	30	3
			Silica	11	
			Trideceth-10	12	
3.	Eyeshadow	United Kingdom	Silica	8	3
			Methylparaben, propylparaben	13, 14	
4.	Eyeshadow	Poland	Silica	8	1
5.	Eyeshadow	Poland	Silica	4	4
			Methylparaben, propylparaben	8, 9	
			BHT (*tert*-butylated hydroxyanisole)	10	
6.	Makeup base	Germany	Methylparaben, propylparaben, butylparaben	11, 13, 16	4
			BHT (*tert*-butylated hydroxyanisole)	21	
7.	Makeup base	Poland	Methylparaben	18	3
			PEG-40 hydrogenated castor oil, PEG-26 buteth-26	11, 12	
8.	Makeup base	United Kingdom	Imidazolidinyl urea, DMDM hydantoin	13, 14	2
9.	Makeup base	France	Methylparaben, butylparaben	15, 16	2
10.	Eyeliner	Germany	CI 77266 (carbon black), black 2	14	4
			Methylparaben, propylparaben	9, 10	
			Beheneth-30 (ethoxylated docosan-1-ol)	4	
11.	Eyeliner	Sweden	Imidazolidinyl urea	13	3
			Methylparaben	12	
			Sorbeth-20 beeswax	6	
12.	Eyeliner	France	CI 77266 (carbon black) [nano]	2	3
			Methylparaben, propylparaben	9, 11	
13.	Eyeliner	Poland	Black 2 (CI 77266)	26	2
			Silica	16	
14.	Concealer	United Kingdom	Silica Silylate	10	6
			BHT (*tert*-butylated hydroxyanisole)	47	
			Cetyl PEG/PPG-10/1 dimethicone, bis-PEG/PPG-14/14 dimethicone, laureth-7, laureth-4	6, 16, 18, 35	
15.	Concealer	Poland	DMDM hydantoin	23	7
			Silica	30	
			Methylparaben, propylparaben	27, 29	
			Cetyl PEG/PPG-10/1 dimethicone, steareth-21, PEG-8	12, 22, 26	
16.	Concealer	France	PEG-10 dimethicone, bis-PEG/PPG-14/14 dimethicon, steareth-20	7, 8, 18	3
17.	Concealer	Italy	Cetyl PEG/PPG-10/1 dimethicone, lauryl PEG/PPG-18/18 methicone	7, 13	2
18.	Concealer	Poland	PEG/PPG-18/18 dimethicone, laureth-7	6, 17	2
19.	Powder/blusher	Poland	Silica	2	4
			Methylparaben, propylparaben	6, 7	
			PEG-8	8	
20.	Powder/blusher	France	Silica	2	3
			Laureth-4, PEG 150 distearate	8, 9	
21.	Powder/blusher	United Kingdom	Methylparaben, propylparaben	6, 7	2
22.	Powder/blusher	Poland	Silica	10	2
			PEG-8	15	
23.	Powder/bronzer	United Kingdom	Methylparaben, ethylparaben, propylparaben, butylparaben	11, 18, 19, 21	5
			BHT (*tert*-butylated hydroxyanisole)	16	
24.	Powder/bronzer	Italy	Imidazolidinyl urea	17	2
			Silica	5	
25.	Powder/bronzer	United Kingdom	Silica	11	3
			Methylparaben, propylparaben	13, 14	
26.	Powder/bronzer	Poland	Methylparaben, ethylparaben, propylparaben	11, 12, 13	4
			PEG-8	14	
27.	Powder/highlighter	United Kingdom	Methylparaben, propylparaben	8, 9	1
28.	Powder/highlighter	Poland	Methylparaben, ethylparaben, propylparaben, butylparaben	13, 14, 15, 16	5
			BHT (*tert*-butylated hydroxyanisole)	17	
29.	Highlighter	United Kingdom	Triethanolamine	16	4
			Diazolidinyl urea	25	
			Methylparaben, propylparaben	24, 26	
30.	Powder	Germany	Silica	13	5
			Methylparaben, ethylparaben, propylparaben	19, 24, 30	
			PEG-150	12	
31.	Powder	Germany	Silica	4	5
			Methylparaben, ethylparaben, propylparaben	23, 24, 25	
			BHT (*tert*-butylated hydroxyanisole)	22	
32.	Lip-tinting product	United Kingdom	Silica	14	4
			Propylparaben	17	
			BHT (*tert*-butylated hydroxyanisole)	22	
			Cetyl PEG/PPG-10/1 dimethicone	12	
33.	Lip-tinting product	Germany	Silica	18	3
			Ethylparaben	17	
			PEG-8	16	
34.	Lip-tinting product	Sweden	Triethanolamine	7	4
			Methylparaben	9	
			PEG-55 propylene glycol oleate, PEG-40 Hydrogenated castor oil	3, 4	
35.	Lip-tinting product	United Kingdom	Silica	4	2
			PEG-45/dodecyl glycol copolymer	10	
36.	Lip-tinting product	Poland	PPG-3 hydrogenated castor oil, PEG-8 tocopherol	1, 24	2
37.	Cosmetic pencil	Poland	BHT (*tert*-butylated hydroxyanisole)	15	2
			PEG-8	13	
38.	Cosmetic pencil	Poland	Methylparaben, propylparaben	21, 22	4
			BHT (*tert*-butylated hydroxyanisole)	23	
			PEG-40 hydrogenated castor oil	7	
39.	Cosmetic pencil	Germany	CI 77266 (carbon black) (nano)	7	2
			PEG/PPG-19/19 dimethicone	3	
40.	Mascara	Poland	CI 77266 (black 2) (nano)	30	4
41.	Mascara	France	CI 77266 black 2	13	3
			Silica	7	
			Methylparaben	5	
42.	Mascara	United Kingdom	Triethanolamine	8	4
			Quaternium 15	12	
			Methylparaben, butylparaben	11, 13	
43.	Mascara	United Kingdom	Silica	22	3
			PEG/PPG-17/18 dimethicone, steareth-20	13, 14	
44.	Mascara	Sweden	Triethanolamine	6	8
			Imidazolidinyl urea	21	
			Silica	13	
			Methylparaben, propylparaben	18, 27	
			BHT (*tert*-butylated hydroxyanisole)	28	
45.	Foundation	France	Silica	20	3
			BHT (*tert*-butylated hydroxyanisole)	31	
			PEG-10 dimethicone	5	
46.	Foundation	France	Imidazolidinyl urea	21	4
			BHT (*tert*-butylated hydroxyanisole)	19	
			PEG/PPG-20/20, laureth-7	7, 16	
47.	Foundation	France	Silica [nano]/silica	11	3
			BHT (*tert*-butylated hydroxyanisole)	17	
			PEG-10 dimethicone, cetyl PEG/PPG-10/1 dimethicone	9, 16	
48.	Foundation	United Kingdom	Silica	24	6
			Methylparaben, butylparaben	17, 20	
			PEG-10 dimethicone, cetyl PEG/PPG-10/1 dimethicone, PEG-20,	7, 8, 9	
49.	Foundation	United Kingdom	Silica	24	6
			Methylparaben, butylparaben	17, 20	
			PEG-10 dimethicone, cetyl PEG/PPG-10/1 dimethicone, PEG-20,	7, 8, 9	
50.	Foundation	United Kingdom	BHT (*tert*-butylated hydroxyanisole)	29	3
			Laureth-4, laureth-30	20, 22	

* The full names of the cosmetics were removed due to the law regulations.

**Table 2:** Potentially carcinogenic substances in cosmetics, together with the potential risks and mechanisms of action.

Ingredient Name	Exposure Route	Potential Risks	The Mechanism	IARC Class	References
Ethanolamines	Transdermal route	Ability to cross the skin barrier; Disruption of cell membrane function and structure; Reports of liver cancer development in mice and organ toxicity (kidney).	Disruption of phospholipid metabolism, resulting in disruption of cell membrane function and structure; Inhibition of choline uptake by liver cells disruption of methylation; Generation of reactive oxygen forms; Formation of nitrosamines.	2B or 3	[27,28,29,30]
Formaldehyde and its donors	Percutaneous route/ respiratory	High absorption rate of formaldehyde from the skin surface; Risk of mutagenicity; Formation of nitrosamines in combination with nitrogen donors; Induction of formation of stable cross-linking between nitrogen bases of DNA nucleotides; Damage to lungs, upper respiratory tract, bone marrow, and brain; Toxicity dependent on the formulation used.	Reduction of miRNA expression; Induction of the formation of stable cross-links between nitrogenous bases of DNA nucleotides.	1 (CH_2_O)	[31,32,33]
Parabens	Percutaneous route	Their level of absorption depends on their chemical structure; The possibility of penetration through the skin and absorption into the bloodstream, transport to organs, and accumulation in adipose tissue; Increased risk of breast cancer in women.	Ability to bind to estrogen receptors; Effect on receptor-controlled genes for estrogen.	not provided	[34,35,36,37,38]
*Tert*-butyl compounds	Percutaneous route	Estrogen-like effects; Proliferative effect on human breast cancer cells.	Inactivation of p53 protein, inhibition of TP53 transcription, release of cytochrome c, activation of caspases, and induction of apoptosis; Receptor affinity; Apoptosis induction; Induction of the formation of reactive oxygen species.	2B (BHA) 3 (BHT)	[39,40,41,42,43]
Ethoxylated compounds	Transdermal/ respiratory route	1,4-dioxane contamination; potential for dioxane to penetrate the skin and exert toxicity; Ethylene oxide contamination with proven carcinogenic effects; Ethylene oxide contamination; reports of lymphatic, hematopoietic, and breast cancers; Histological changes involving the liver and kidneys; Increased incidence of liver cancer (rats); Risk of breast cancer (ethylene oxide); Evidence from animal studies is “sufficient” and “extensive” for EtO.	Influence on the activity of ribonucleic acid polymerases, causing chromosome aberrations, including deletions; Possibility of forming DNA adducts.	1 (EtO) 2B (1,4-dioxane)3 or not provided	[44,45,46,47,48,49,50,51]
Arsenic	Percutaneous route	Skin discoloration; Development of precancerous conditions and skin cancers (Bowen’s disease and basal cell carcinoma); Reports of the development of lung cancer; Accumulation in keratin-rich tissues such as skin, nails, and hair. Tumor agent in cancers of the breast, lung, and other organs.	Stimulation of cancer cell proliferation; Creation of reactive oxygen species; Induction of oxidative stress; DNA damage.	1	[52,53,54,55,56]
Cadmium	Transdermal/ respiratory route	Possibility of penetration through the skin; Reports of accumulation in the liver and kidneys; Liver and kidney dysfunction; Reports of development of lung, prostate, prostate, kidney, testicular, and breast cancer.	Stimulation of oxidative stress; Disruption of intercellular signaling; Reduction in the activity of antioxidant enzymes and DNA repair enzymes; Formation of reactive oxygen species; Disruption of cell signaling; Effect on DNA methylation.	1	[57,58,59,60,61,62,63,64]
Lead	Transdermal/ respiratory route	Possibility of penetration through the skin; Reports of accumulation in organs; Reports of the development of lung and stomach cancers in humans Nephrotoxicity (risk of kidney cancer).	Stimulation of endothelial cells and Langerhans cells to produce post-inflammatory cytokines; Formation of free radicals; Induction of oxidative stress; Reduction in the activity of antioxidant and DNA repair enzymes.	2B	[57,65,66,67]
Mercury	Percutaneous route	Ability to penetrate the skin and its appendages; Induction of lung tumors.	Stimulation of production of pro-inflammatory cytokines; Genetic damage to cells, stimulation of pro-inflammatory cytokine production; Pro-oxidant factor.	2B	[25,68,69,70,71,72,73,74]
Carbon black	Transdermal/ respiratory route	Redness of the conjunctiva of the eyelids; Development of cutaneous squamous cell carcinomas and skin papillomas; atrophy and fibrosis of the skin.	Lymphocytic infiltration, pigment uptake by macrophages.	2B	[75,76,77,78,79,80]
Silica	Transdermal/ respiratory route	Possibility of skin penetration of silica nanoparticles (>100 nm); Cytotoxicity; Pulmonary fibrosis; Risk of carcinogenicity.	Reactive oxygen form production, cellular DNA damage; Activation of macrophages, pro-inflammatory cytokines, development of inflammation, and production of free radicals.	1	[81,82,83,84,85]

**Table 3:** Chemical structure of ethanolamines and their derivatives.

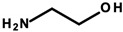	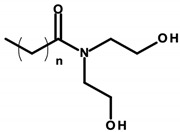 n = 6, 8, 10, 12, 14, 16
2-aminoethanol (IUPAC) ethanolamine	Cocamides Diethanolamides N,N-bis(2-hydroxyethyl)octanamide (n = 6) N,N-bis(2-hydroxyethyl)decanamide (n = 8) N,N-bis(2-hydroxyethyl)dodecanamide (n = 10) N,N-bis(2-hydroxyethyl)tetradecanamide (n = 12) N,N-bis(2-hydroxyethyl)hexadecanamide (n = 14) N,N-bis(2-hydroxyethyl)octadecanamide (n = 14)
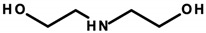	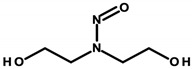
Diethanolamine (DEA) 2-(2-hydroxyethylamino)ethanol	N-nitrosodiethanolamine (NDELA) 2-[(2-hydroxyethyl)(nitroso)amino]ethan-1-ol
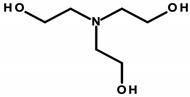	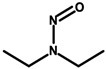
Triethanolamine (TEA), 2,2′,2″-nitrilotriethanol 2-(bis(2-hydroxyethyl)amino)ethanol	N-nitrosodiethylamine (NDEA) Diethylnitrous amide N-ethyl-N-nitrosoethanamine

**Table 4:** Chemical structure of formaldehyde donors.

Quaternium-15 1-(3-chloroallyl)-3,5,7-triaza-1-azoniaadamantane chloride	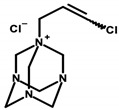
DMDM hydantoin 1,3-Bis(hydroxymethyl)-5,5-dimethylimidazolidine-2,4-dione	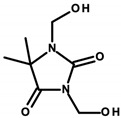
Imidazolidinyl urea 1,1’-methylenebis{3-[4-(hydroxymethyl)-2,5-dioxoimidazolidin-4-yl]urea}	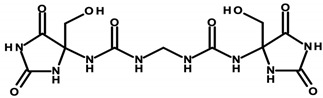
Sodium N-(hydroxymethyl)glycinate	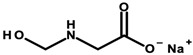
Polynoxylin (poly) methylene-N,N’-bis(hydroxymethyl)urea	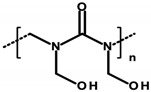
Diazolidinyl urea 1-[3,4-bis(hydroxymethyl)-2,5-dioxoimidazolidin-4-yl]-1,3-bis(hydroxymethyl)urea	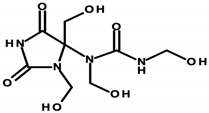
Glyoxal oxalaldehyde	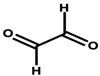
Bronopol 2-bromo-2-nitropropane-1,3-diol	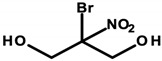
Bronidox 5-bromo-5-nitro-1,3-dioxane	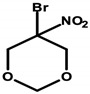

**Table 5:** Chemical structure of parabens.

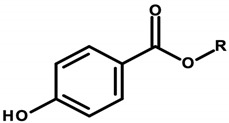	R = CH3 R = CH2CH3 R = CH(CH3)2 R = (CH2)2CH3 R = CH2CH(CH3)2 R = (CH2)3CH3 R = CH2C6H5	methylparaben ethylparaben isopropylparaben propylparaben isobutylparaben butylparaben benzylparaben
Paraben *para*-hydroxybenzoate 4-hydroxybenzoate		

**Table 6:** Chemical structure of *tert*-butyl compounds.

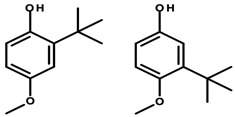	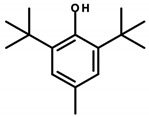
BHA, butylated hydroxyanisoles 2-*tert*-butyl-4-hydroxyanisole, 3-*tert*-butyl-4-hydroxyanisole 2-*tert*-butyl-4-methoxyphenol and 3-*tert*-butyl-4-methoxyphenol (IUPAC)	BHT, butylated hydroxytoluene 2,6-di-*tert*-butyl-4-methylphenol

**Table 7:** Chemical structure of ethoxylated compounds.

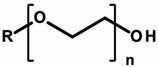	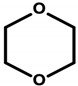
Laureth, polyethylene glycol monododecyl ether, ethoxylated dodecanol (lauryl alcohol), R = CH3(CH2)11 Steareth, polyethylene glycol monooctadecyl ether, ethoxylated octadecanol (stearyl alcohol), R = CH3(CH2)17 Ceteth, polyethylene glycol monohexadecyl ether, ethoxylated hexadecanol (cetyl alcohol), R = CH3(CH2)15	1,4-dioxane
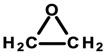	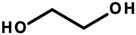
Ethylene oxide Oxirane, epoxyethane	ethylene glycol ethane-1,2-diol

## Data Availability

Data available within article.
